# Redefining the gender identity spectrum in longitudinal studies: adolescent response patterns after adopting the two-step measure of sex and gender

**DOI:** 10.24095/hpcdp.45.11/12.04

**Published:** 2025

**Authors:** Thepikaa Varatharajan, Angelica Amores, Karen A. Patte, Margaret de Groh, Ying Jiang, Scott T. Leatherdale

**Affiliations:** 1 School of Public Health Sciences, University of Waterloo, Waterloo, Ontario, Canada; 2 Centre for Surveillance and Applied Research, Health Promotion and Chronic Disease Prevention Branch, Public Health Agency of Canada, Ottawa, Ontario, Canada; 3 Department of Health Sciences, Brock University, St. Catharines, Ontario, Canada

**Keywords:** adolescents, Canadian, gender identity, longitudinal analysis, population health, two-step measure, sex, surveillance systems

## Abstract

Ongoing, large-scale longitudinal studies and surveillance systems are moving beyond historical single-item sex or gender measures to better capture gender identity. We examined patterns in adolescents’ responses over a two-year period (2020–2021 to 2021–2022 school years) after the COMPASS study adopted a two-step measure of gender identity. Descriptive analyses revealed that, over time, 3.5% and 5.5% of high school students (n=11618) selected a different response for sex and gender, respectively. Our findings show that by implementing an inclusive measure that recognizes sex and gender as distinct constructs can improve the identification of all gender identities without compromising data quality.

**Highlights**

- Historical single-item measures often do not differentiate between sex and gender and/or provide only binary response options (e.g. “male” and “female”).
- This study examined patterns in adolescents’ responses regarding their sex and gender after an ongoing longitudinal study adopted a twostep measure of gender identity.
- We found that over a two-year period, 3.5% of adolescents selected a different response for sex (e.g. “female” to “male”) and 5.5% selected a different response for gender (e.g. “female” to “boy”).
- Collecting more inclusive data on gender identity in population-based longitudinal studies can fill data gaps, identify gender-related health disparities and strengthen the health and well-being of all Canadians.

## Introduction

The scarcity of reliable and comprehensive epidemiological data on Canadians’ gender identity has hindered the understanding of health disparities among cisgender, transgender and gender-diverse individuals.^1^,^2^ This is primarily because large-scale population-based health surveys assess gender identity using a single question (e.g. “Are you male or female”). Using a measure of sex as a proxy for measuring gender identity conflates concepts of sex and gender and limits responses to traditional binary options.^3^,^4^ To improve inclusivity and address the existing data gaps, Statistics Canada adopted a new standard for measuring sex and gender.^5^ This initiative, along with the recent calls for national organizations, funding agencies and journal editors to conduct sex- and gender-based analyses, has prompted researchers to improve data collection of individuals’ sex and gender identity.^1^,^2^

Specifically, ongoing large-scale population-based studies and surveillance systems are encouraged to improve their assessment of sex and gender identity.^2^,^6^ School-based studies such as the Canadian Student Alcohol and Drugs Survey^7^ and the COMPASS (Cannabis, Obesity, Mental health, Physical Activity, Sedentary behaviour and Smoking) study^8^ have adopted a valid and cognitively feasible two-step gender identity measure to assess and differentiate between sex assigned at birth (SAB; Step 1) and gender identity (Step 2).^6^,^9^ Distinguishing SAB from gender identity has been shown to improve the accuracy of identifying cisgender, transgender and gender-diverse people; to increase population-level prevalence estimates of all gender identities; and to improve knowledge of health disparities among people with different gender identities.^2^,^3^,^9^-^13^

While evidence supports adopting an inclusive measure of gender identity,^9^-^13^ modifying relevant questions in longitudinal surveys could result in inconsistencies in coding gender identity, affecting the assessment of trends or changes over time.^14^ To our knowledge, no study has explored the prospective effects of changing a key demographic variable in an ongoing longitudinal study. Therefore, the goal of this study was to examine patterns in adolescents’ responses to sex and gender identification questions after switching from a one-step to a two-step gender identity measure.

## Methods

***Ethics approval***

All COMPASS procedures were approved by the University of Waterloo Office of Research Ethics (ORE# 30118) and participating school boards.

***Data source***

We utilized two-year linked student-level data from Year 9 (Y9; 2020–2021) and Year 10 (Y10; 2021–2022) of the COMPASS study to examine patterns in adolescents’ responses to questions about their sex and gender. COMPASS is a 12-year, prospective, hierarchal cohort study designed to collect self-reported health data annually from a sample of students (Grades 9–12; Secondary I–V in Quebec) and their secondary schools.^8^

A total of 11746 students from 123 schools (Alberta,n =5; British Columbia, n=11; Ontario, n =50; and Quebec, n=57) participated in the study across the 2 years. An online survey was used to collect student-level data during the school year (September to June). Schools that permitted active-information passive-consent data collection protocols^15^ emailed a survey link to eligible students (i.e. all students attending participating schools who were not withdrawn from the study by their parent or guardian). Surveys were completed either during class time or at home. Detailed study methods are described elsewhere.^15^,^16^

***Measures***

**Sex/gender**

Consistent with measures used in youth surveillance systems during data collection,^7^,^17^ students in Y9 were asked, “Are you female or male?” Response options were “female,” “male,” “I describe my gender in a different way” or “I prefer not to say.” Given the unclear distinction between biological sex and social gender identity, we refer to this measure as assessing sex/gender.^17^

**SAB and gender identity**

In Y10, students were asked two questions: (1)“What sex were you assigned at birth?” (response options were “female,” “male” and “I prefer not to say”); and (2)“Which gender do you most identify with?” (response options were “girl/woman,”* “boy/man,”* “Two Spirit,” “nonbinary person,” “I describe my gender differently” or “I prefer not to say”). 

The measures used for Y10 students demonstrate validity for adolescents.^3^,^12^,^18^

***Data linkage***

The standard COMPASS data linkage process involves generating a unique six-digit code for each student.^19^ This code is based on consistent responses to five specific questions asked at the beginning of the questionnaire (e.g. last letter of your full last name) plus the sex/gender question. Since sex/gender is an essential study measure, we removed it from the linkage process. To ensure that the new data linkage method was robust, we only included individuals who were linked consistently in both the old^19^ and new data linkage methods (n=11746). Links found exclusively in the new linkage method (n=189) were excluded to reduce potential false links.

***Analysis***

All analyses were conducted in SAS version 10.1 (SAS Institute Inc., Cary, NC, US).^20^ Descriptive statistics were conducted to explore patterns of responses. We used McNemar tests to compare responses over time.

Students with missing responses to the Y9 sex/gender measure, the Y10 SAB measure and/or the Y10 gender identity measure (n=101) were removed from the sample. Due to ethical considerations regarding Indigenous populations, we refrained from reporting on participants who identified as Two Spirit. 

*For simplicity, the response options have been abbreviated to “girl” and “boy.”

## Results

Our sample comprised 11 618 students (aged 12–19 years, with the majority identifying as White). In Y9, 6523 reported their sex/gender as female and 4748 as male; 198 described their gender differently and 115 preferred not to say.

**Figure 1:**
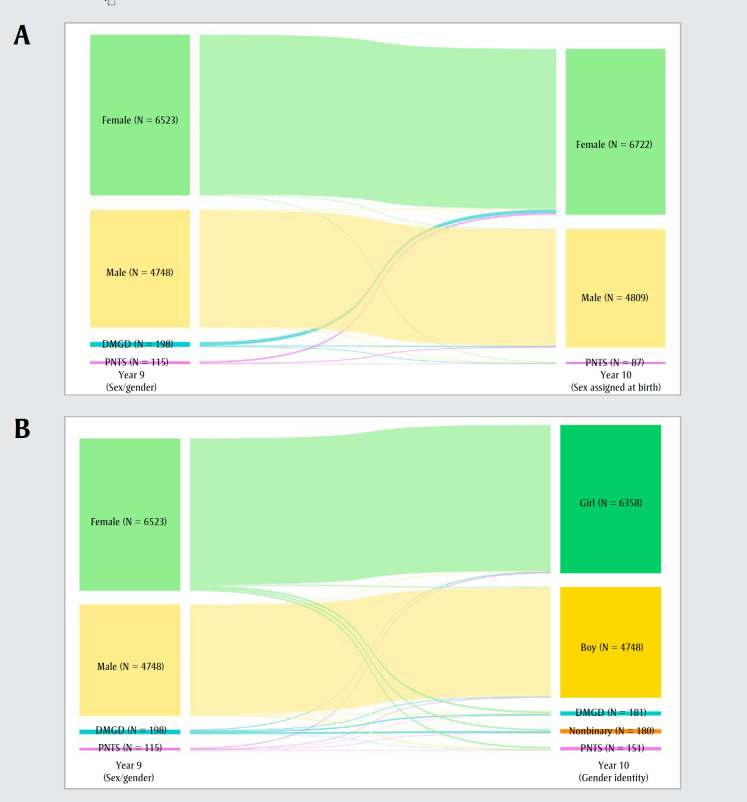
Adolescents’ (12–19 years) response patterns to sex/gender, sex assigned at birth and gender identity questions, from Y9 (2020–2021) to Y10 (2021–2022), COMPASS study, n = 11 618, Canada

**Abbreviations:** DMGD, describe my gender differently; PNTS, prefer not to say; Y9, Year 9; Y10, Year 10. 

**Notes: **Figure 1A depicts the proportion of students responding in Y9 (2020–2021) to sex/gender (“Are you female or male?” with response options “female,” “male,” “I describe my gender differently” [DMGD] or “I prefer not to say” [PNTS]) and in Y10 (2021–2022) to sex assigned at birth (“What sex were you assigned at birth?” with response options “female,” “male” and “I prefer not to say” [PNTS]). Figure 1B depicts the proportion of students responding in Y9 (2020–2021) to sex/gender (“Are you female or male?” with response options “female,” “male,” “I describe my gender in a different way” [DMGD] or “I prefer not to say” [PNTS]) and in Y10 (2021–2022) to gender identity (“Which gender do you most identify with?” with response options “girl/woman” [shown as “girl”], “boy/man” [shown as “boy”], “Two Spirit” [not reported for ethical considerations regarding Indigenous populations], “nonbinary person,” “I describe my gender differently” [DMGD] or “I prefer not to say” [PNTS]). The Figure 1B bars for Y10 gender identity responses “girl” and “boy” are slightly darker in colour to differentiate them from Y9 sex/gender responses “female” and “male.” The size of the coloured bars represents the proportion of students within each category, with thicker bars representing a higher proportion of students. 

In Y10, 6722 students reported their SAB as female and 4809 as male; 87 preferred not to say. In response to the Y10 gender identity measure, 6358 students identified as a girl, 4748 as a boy and 180 as nonbinary; 181 described their gender differently; 151 preferred not to say (Figure 1).

***Response patterns across time***

The proportion of adolescents who selected “female” (56.1% vs. 57.9%; *p*<0.0001) or “male” (41.2% vs. 41.4%; *p*=0.02) increased over time (percentages in Table 1 have been summed). A significantly higher percentage selected “female” (55.8%) or “male” (40.6%) in Y9 than those who selected “girl” (54.7%) or “boy” (40.9%) in Y10 (*p*<0.0001). The number of adolescents who selected a response option indicating gender diversity (i.e. “I describe my gender differently” or “nonbinary person”) also increased (1.7% to 3.4%; *p*<0.0001). For instance, 1.6% selected “nonbinary person” in Y10, regardless of their response in Y9.

**Table 1:** Sex and gender identity measure responses by adolescents (12–19 years) in Y9 (2020–2021) and Y10 (2021–2022) of the COMPASS study, n = 11 618, Canada

Year 9 responses	Year 10 responses	Frequency, n	Proportion, %
Sex/gender to sex assigned at birth			
Female	Female	6479	55.8
Female	Male	23	0.2
Female	PNTS	21	0.2
Male	Male	4722	40.6
Male	Female	28	0.2
Male	PNTS	32	0.3
DMGD	Female	144	1.2
DMGD	Male	31	0.3
DMGD	PNTS	23	0.2
PNTS	Female	71	0.6
PNTS	Male	33	0.3
PNTS	PNTS	11	0.1
Sex/gender to gender identity			
Female	Girl	6260	53.9
Female	Boy	41	0.4
Female	DMGD	79	0.7
Female	Nonbinary	69	0.6
Female	PNTS	74	0.6
Male	Boy	4651	40.0
Male	Girl	26	0.2
Male	DMGD	38	0.3
Male	Nonbinary	19	0.2
Male	PNTS	48	0.4
DMGD	DMGD	50	0.4
DMGD	Boy	29	0.2
DMGD	Girl	34	0.3
DMGD	Nonbinary	74	0.6
DMGD	PNTS	11	0.1
PNTS	PNTS	18	0.2
PNTS	Girl	38	0.3
PNTS	Boy	27	0.2
PNTS	DMGD	14	0.1
PNTS	Nonbinary	18	0.2

**Abbreviations:** DMGD, describe my gender differently; PNTS, prefer not to say; Y9, Year 9; Y10, Year 10. 

When we compared the Y9 sex/gender responses to the Y10 SAB responses, we found that 11 212 (96.5%) adolescents selected the same response, while 406 (3.5%) selected a different response (e.g. “female”, “describe my gender differently” or “prefer not to say” to “male”) (Table 1). When we compared the Y9 sex/gender responses to the Y10 gender identity responses, we found that 10979 adolescents (94.5%) selected a response where their sex likened to their gender identity (e.g. “female” to “girl”), while 639 (5.5%) selected a different response (e.g. “female”, “describe my gender differently” or “prefer not to say” to “boy”).

***Adolescents’ current gender identity***

Based on the Y10 SAB and gender identity responses, we categorized the participating students as cisgender girls (n=6331); cisgender boys (n=4688); transgender and gender-diverse adolescents (n=385); and prefer not to answer (n=214). We determined that there were 321 transgender and gender-diverse adolescents directly, based on their gender identity responses (nonbinary, n =156; “I describe my gender differently,” n=165), and 64 indirectly, based on the incongruence between their self-reported SAB and gender identity.

## Discussion

The goal of this study was to examine whether adopting a more inclusive measure of gender identity in the ongoing COMPASS study influenced patterns in the participating students’ responses to sex and gender identity over time. Our results indicate that the response patterns for each construct remained largely comparable, with only small proportions of participants choosing different responses. Therefore, expanding the gender identity measure in a population-based longitudinal survey can provide additional insights without compromising data quality.

There may be several reasons why participants changed their responses. Compared to the sex/gender measure, the wording, intent and order of each two-step measure question may have been easier for the surveyed adolescents to understand.^13^,^21^ This may also explain why the majority of the sample had overlapping responses for sex/gender in Y9 and SAB in Y10. Asking about SAB first may provide context to the question about gender identity, particularly for cisgender adolescents who, unlike their transgender and gender-diverse peers, may be unaccustomed to thinking of gender as an identity.^11^,^22^ The additional response options offered in Y10 could have also helped respondents choose an answer that best describes their gender identity.^10^ For instance, among participants, approximately 0.6% selected “I describe my gender differently” in Y9 and then opted to select “nonbinary” in Y10. 

Evidence from prior research indicates that gender identity is a fluid construct that follows no specific pattern.^23^,^24^ Although we cannot be certain how participants interpreted the Y9 sex/gender measure (i.e. they may have responded referring to either their sex or their gender), it is plausible that some modified their responses because of changes in their gender identity (i.e. the respondents may have been exploring or questioning their gender identity).^12^ Katz-Wise et al.^23^ found that 28.9% of transgender and gender-diverse adolescents (N=183; aged 14–17 years) changed their gender identity after 1.5 years, with an equal number transitioning toward a binary and a nonbinary gender identity. Thus, it is important for longitudinal studies to repeatedly collect SAB and gender identity data, as doing so allows participants to accurately update their gender identity and informs researchers about the fluidity of participants’ gender identity.^2^,^12^ If a longitudinal study has inconsistent measures at different time points, Bailey et al. recommended reporting the most recent data, thereby preventing measurement error and the misapplication of research findings.^2^

***Strengths and limitations***

This study benefits from utilizing data from COMPASS, Canada’s largest ongoing, school-based study (2012–2027). COMPASS employs a prospective study design and can achieve a large sample size.

Nevertheless, our study has limitations. First, COMPASS is not designed to be representative, although the use of whole-school samples and the positive response rates strengthens generalizability. Second, self-reported measures are subject to social desirability bias. However, the use of passive-consent procedures and anonymous IDs has been shown to reduce selection bias and encourage open, honest responses.^15^

## Conclusion

Gender identity is an important social determinant of health that influences health outcomes, behaviours and service use, particularly among transgender and gender-diverse people.^13^ As adolescence is a key period during which gender identity is explored, it is important that health surveys targeting secondary school students differentiate between SAB and gender identity using validated and reliable measures.^12^ Adopting a measure that is inclusive of gender identity in longitudinal studies will improve representation for all gender identities, particularly transgender and gender-diverse individuals, and highlight their unique needs to policy makers, school personnel and other decision makers who depend on reliable data from population-based health surveys. 

## Acknowledgements

The COMPASS study has been supported by a bridge grant from the Canadian Institutes of Health Research (CIHR) Institute of Nutrition, Metabolism and Diabetes through the “Obesity – Interventions to Prevent or Treat” priority funding awards (OOP-110788; awarded to STL); an operating grant from the CIHR Institute of Population and Public Health (IPPH) (MOP-114875; awarded to STL); a CIHR project grant (PJT-148562; awarded to STL); a CIHR bridge grant (PJT-149092; awarded to KAP/STL); a CIHR project grant (PJT-159693; awarded to KAP); a research funding arrangement with Health Canada (#1617-HQ-000012; contract awarded to STL), a CIHR-Canadian Centre on Substance Use and Addiction team grant (OF7 B1-PCPEGT 410-10-9633; awarded to STL); and aproject grant from the CIHR IPPH (PJT-180262; awarded to STL and KAP). The COMPASS-Quebec project also benefits from funding from the Ministre de la sant et des services sociaux of the province of Quebec and the Direction rgionale de sant publique du CIUSSS de la Capitale-Nationale. KAP is the Tier II Canada Research Chair in Child Health Equity and Inclusion. TV is funded by the Ontario Graduate Scholarship and by the Public Health Agency of Canada through the Federal Student Work Experience Program.

## Conflicts of interest

MdG is a former Associate Editor-in-Chief of the *HPCDP* Journal and STL is a former Associate Scientific Editor. Both recused themselves from the review process for this article.

The authors declare that they have no conflicts of interest.

## Authors’ contributions and statement

TV: Conceptualization, methodology, formal analysis, data curation, writing—original draft, writing—review and editing.

AA: Conceptualization, methodology, data curation, writing—review and editing.

KAP: Supervision, data curation, funding acquisition, resources, writing—review and editing. 

MdG: Supervision, conceptualization, methodology, resources, writing—review and editing.

YJ: Supervision, conceptualization, methodology, resources, writing—review and editing.

STL: Supervision, data curation, funding acquisition, resources, conceptualization, methodology, investigation, writing—review and editing.

The content and views expressed in this article are those of the authors and do not necessarily reflect those of the Government of Canada.
